# The effect of midwifery-led continuum of care to improve maternal and newborn outcomes in the Sidama region, Ethiopia: A non-randomized control trial study

**DOI:** 10.1177/20503121251383995

**Published:** 2025-11-05

**Authors:** Rekiku Fikre, Sanne Gerards, Wondwosen Teklesilasie, Jessica Gubbels

**Affiliations:** 1 Department of Health Promotion, Faculty of Health, Medicine & Life Sciences, NUTRIM Institute of Nutrition and Translational Research in Metabolism, Maastricht University, The Netherlands; 2 Department of Midwifery, College of Medicine and Health Sciences, Hawassa University, Ethiopia; 3 School of Public Health, College of Medicine and Health Sciences, Hawassa University, Ethiopia

**Keywords:** Midwifery-led continuum care, Ethiopia, maternal outcomes, neonatal outcomes, nonrandomized control trial

## Abstract

**Background:**

The midwifery-led continuum of care model is an approach where a single midwife or a team of midwives provides comprehensive care to a woman throughout pregnancy, childbirth, and the early postpartum period. This model of care has significantly improved maternal and newborn outcomes; however, it is primarily implemented in high-income countries.

**Objectives:**

To evaluate the effect of the midwifery-led continuum of care model on maternal and neonatal health outcomes in general hospitals of the Sidama region in Ethiopia.

**Methods:**

A prospective nonrandomized control trial was conducted from October 2023 to June 2024 in the Sidama region, Ethiopia. A systematic sampling technique was used to recruit 478 low-risk women in total for the intervention group and control group. Multivariable analysis for binary outcomes with the log link were conducted to estimate adjusted risk ratios Adjusted risk ratio (aRR) and 95% confidence intervals.

**Results:**

Women in the intervention were significantly more likely to have spontaneous vaginal birth (aRR) of 1.21 (95% confidence interval 1.14–1.67), and less likely to have preterm birth aRR of 0.16 (95% confidence interval 0.11–0.57) in comparison with women and newborns who received shared care.

**Conclusion:**

Women in the intervention group experience improved outcomes and seems to be a valuable strategy for improving pregnancy outcomes in low-resource settings. Further research should enhance the practical application of midwifery-led continuum of care for women facing social risk factors, and medical complications in low-resource settings.

**Trial registration:**

PACTR202310532830947

**Website:**

https://pactr.samrc.ac.za/

## Introduction

Globally, maternal and newborn mortality remain a significant public health problem.^1^ In 2020, ~287,000 women died during pregnancy and childbirth, and around 6500 newborns lost their lives.^1,2^ The burden is especially high in low-resource settings, which account for nearly 95% of all global maternal mortalities.^1^ Specifically, sub-Saharan Africa contributes to about 70% of global maternal deaths, and faces the highest neonatal mortality rate worldwide, with 27 deaths for every 1000 live births.^1,2^

The sub-Saharan low-income country Ethiopia has achieved notable advancements in lowering maternal and neonatal mortality over the past two decades.^3^ Maternal mortality in Ethiopia decreased substantially from 871 in 2000 to 412/100,000 live births by 2016.^4^ Similarly, neonatal mortality has seen a marked improvement, reduced from 49 deaths in 2000 to 30 deaths/1000 live births in 2019.^5^ Despite these encouraging trends, Ethiopia still lags behind on global targets for mortality reduction of 199 maternal deaths/100,000 live births and 10 neonatal deaths/1000 live.^3–5^ Therefore, it is crucial to implement additional interventions to accelerate the reduction of maternal and neonatal mortality.^6^

The World Health Organization has prioritized enhancing midwives’ capacity to provide high-quality maternal and neonatal health services.^7^ The organization advocates the introduction of midwifery-led continuum of care (MLCC) models for pregnant women, especially in regions where midwifery education and training are well established.^8,9^ MLCC is an approach where a single midwife or a team of midwives oversees a woman’s whole pregnancy through childbirth and the early postpartum period.^10^ This comprehensive model ensures continuity of care, while maintaining the flexibility to seek expert advice or transfer care to other healthcare professionals when needed.^10,11^

Evidence showed that MLCC enhances the quality of care, improves maternal and newborn outcomes, contributes to reductions in maternal and neonatal mortality, and minimizes unnecessary interventions.^8,12–14^ A systematic review and evidence from randomized controlled trials showed that women who receive MLCC are likely to experience fewer cesarean sections, more spontaneous vaginal births, less premature births, less instrumental deliveries, less episiotomies, and less premature rupture of membranes.^10,11,15^ However, most of this evidence stems from high-income countries.^13,16^ There is limited evidence suggesting that the MLCC model can be implemented in low-resource settings.^15,17^

Implementing interventions like MLCC has great potential to improve outcomes in regions with high maternal and newborn mortality rates and limited resources,^18,19^ such as Ethiopia. Therefore, this study aimed to evaluate the effect of the MLCC model on maternal and neonatal health outcomes in general hospitals of the Sidama region in Ethiopia.

## Methods

### Study design and setting

A prospective nonrandomized control trial was conducted from October 2023 to June 2024 in four general hospitals of the Sidama region, Ethiopia. The study compares MLCC to shared care, based on maternal and newborn outcomes. The data were collected from December 2023 to June 2024. The Sidama region is organized into 36 districts with Hawassa city serving as the city administration. Sidama is bordered in the south by the Oromia region, in the west by the Wolayita Zone, and in the north and east by the Oromia region. Sidama has an estimated total population of 4,469,455.^20^ The region comprises 21 governmental hospitals, 138 health centers, and 553 health posts serving around five million population. Patients range from those needing basic preventive and outpatient services at health posts and health centers to more complex care requiring inpatient and specialized services at general and specialized hospitals. Health professionals include health extension workers at posts, nurses, midwives, general practitioners at primary and general hospitals, and specialists at the comprehensive hospital. For this study, four hospitals were randomly selected to represent the hospital settings. Each hospital employs ~20–25 midwives and an obstetrician who play a critical role in delivering essential comprehensive obstetric service services, including antenatal care (ANC), skilled birth attendance, and postnatal care. These hospitals serve diverse patient populations, primarily pregnant women requiring comprehensive maternal and newborn care. The range of health professionals includes midwives, nurses, doctors, and specialists, each contributing their expertise to different stages of care.

### Eligibility criteria

MLCC is based on the premise that pregnancy and birth are normal life events.^11^ By including only low-risk women, the model can be implemented as intended, focusing on the midwife’s role in supporting natural birth processes.^11^

#### Inclusion criteria

Women in the selected hospitals who were <24 weeks pregnant during their initial ANC visit and had a low obstetric risk (single pregnancy, term pregnancy, no previous maternal complications, and absence of preexisting medical and obstetric complications) were included for the study.

#### Exclusion criteria

Those with a history of medical or obstetric complications based on the national ANC guideline,^21^ as well as those planning to schedule with another care provider then midwives (nurses, health officers, emergency surgeons, general practitioners, and obstetricians) were excluded from the study.

### Sample size determination

To calculate the appropriate sample size, effect sizes of a study conducted in another region in Ethiopia (North Shoa) on the effects of MLCC on maternal and neonatal health outcomes was used.^15^ This particular study found that the percentage of preterm births in the intervention group was 6.4%, while the control group had a rate of 15.2%.^15^ With a two-sided confidence level of 95% confidence interval (CI) and a power of 80%, the number of mothers receiving MLCC and receiving shared care and assuming a 10% loss to follow-up, the total final sample size was 478 low-risk pregnant women (single pregnancy, term pregnancy, no previous maternal complications, and absence of preexisting medical and obstetric complications) with 239 participants in the control hospitals (shared care) and 239 in the intervention hospitals (MLCC). We used G*Power to calculate the sample size which is developed and maintained by the Psychological Institute at Heinrich Heine University Düsseldorf, Germany.^22^

### Selection procedure and sampling techniques

A two-stage stratified cluster sampling technique was used. In the first stage, four general hospitals (Adare, Leku, Yirgalem, and Bona) in the Sidama region were randomly selected for the study. Then, the calculated samples were equally distributed for each hospital. All the hospitals have a similar governance structure and provide comparable services based on the same care standard and protocols, including offering them free of charge.^23^ Hospitals with midwives willing to participate in MLCC were chosen as intervention sites (Leku and Bona hospital), and the remaining hospitals were taking as control hospitals. The calculated sample size was proportionally distributed among the four hospitals. To estimate the number of pregnant women seeking services, we analyzed ANC data from one quarter for each hospital.

**Figure 1.**
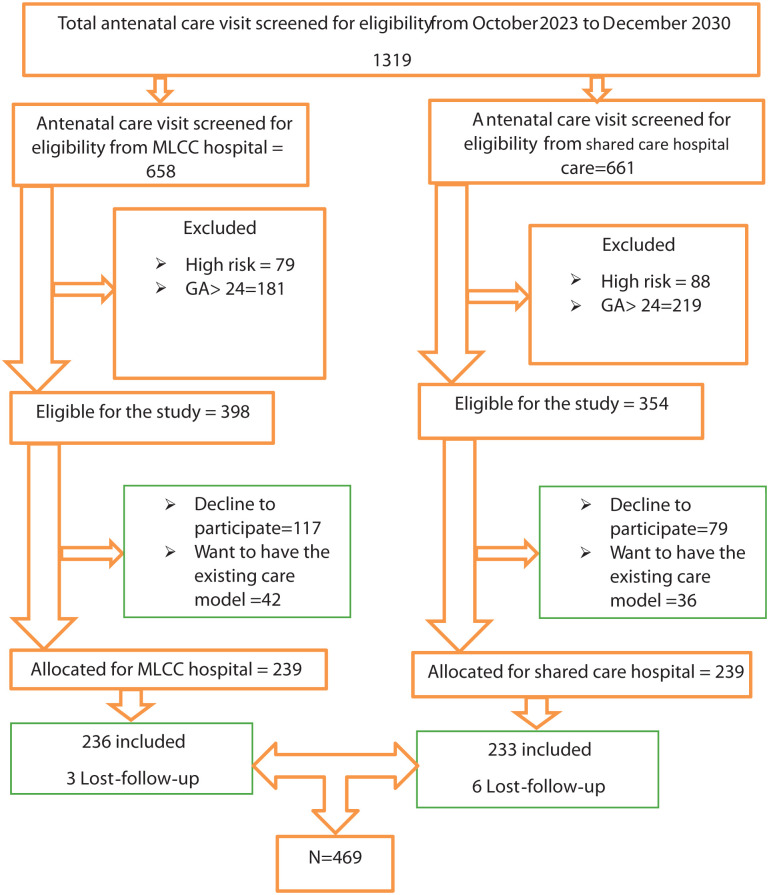
Study participant’s recruitment process from October 2023 to June 2024, in Sidama region, Ethiopia 2024. ANC: antenatal care; GA: gestational age; MLCC: midwifery-led continuum of care.

Then a systematic random sampling technique with a sampling interval of two was used to select the study participants. In each hospital, the first participant was selected by a lottery method. Participants were assigned to either the intervention or control group based on predetermined criteria instead of true randomization. The midwives in the research team explained the aim of the study to the women and screened pregnant women to identify those with low-risk pregnancies based on predefined clinical criteria. These midwives assessed eligibility during antenatal visits and then enrolled eligible women into either the intervention group (receiving MLCC) or the control group (receiving standard shared care). The eligibility of the women was assessed, and then, women were recruited to study by research midwives when they attended their first visit at the antenatal clinic and were allocated to the intervention or control groups at the designated hospitals after written consent had been obtained. Eligible women who attended their first ANC visits at the selected hospitals during the recruitment period, and who met the inclusion criteria, were consecutively enrolled until the required sample size was reached for both groups. To avoid information contamination, the intervention and control hospitals were geographically separated. During the study period, a total of 1319 mothers visited four ANC clinics. Out of these, 624 women were found to be eligible for inclusion in the study. However, 115 women chose not to participate, and 31 preferred to give birth with other health professionals. In total, 478 low-risk women participated in the study, with 239 assigned to the MLCC group and 239 to the shared care group. In the data analysis, 236 women remained in the MLCC group, as three were lost to follow-up, and 233 remained in the shared care group, with six lost to follow-up (see Figure 1). Women who met the inclusion criteria and expressed a willingness to participate were selected until the desired sample size was reached in both groups.

### Intervention group

Women participating in the intervention hospitals received comprehensive MLCC throughout their pregnancy, labor, delivery, and immediate postpartum period. The midwives delivering this care were recruited from two hospitals as part of the intervention to ensure continuous support in the intervention group. These midwives received specialized training for 1 week, focusing on an approach that prioritizes continuity of care and equips them to support women throughout their maternity journey in accordance with the MLCC model.^10^ In this group, the midwives were responsible for ensuring continuous care for each woman and supported one another in the event of a primary midwife’s absence. In MLCC, if complications arise, the primary midwife works in collaboration with other healthcare professionals to ensure continued support.^10^ One midwife from each MLCC team was assigned to the labor ward to offer care during childbirth. Care during labor and delivery was provided in the labor ward of the intervention hospitals in 8-h shifts. If a laboring woman’s care extended beyond 8 h, the midwife would transfer responsibility to another midwife within the team. In case of women undergoing cesarean sections, midwives extended their care to the operating theater. If two women assigned to the same midwife went into labor simultaneously, the midwife could either request assistance from another midwife on the team or the midwives in the labor ward assisted the primary midwife. Following the delivery, the MLCC model continued into the postnatal ward. Each day, one midwife from each team was assigned to the postnatal ward, providing continuity of care for mothers and newborns. This arrangement ensured that women received individualized support, monitoring, and guidance from a familiar midwife who understood their individual needs and preferences throughout their hospital stay, promoting effective recovery and optimal infant care after childbirth.

### Control group

Pregnant women who received antenatal, labor, birth, and postnatal care according to the established practice in Ethiopia. In this group, care was provided according to the shared care model, which involves a distribution of responsibilities among various obstetric care providers.^24^ In this model, responsibility for care is distributed among various healthcare providers, including midwives, nurses, health officers, and doctors. Care is delivered through conventional 8-h shifts, with healthcare providers handing over patients to the next shift’s staff.

After discharge, each woman in this group received care from a different team of midwives or nurses who were assigned in family planning and immunization unit. This approach results in fragmented care, with multiple providers involved at different stages, limiting continuity and personalized support throughout the maternity period. The SMC model contrasts with MLCC, where a single provider or small team offers continuous support from pregnancy through postpartum.

### Outcome variables

Spontaneous vaginal birth (yes/no) and preterm birth were the primary maternal and neonatal outcomes. Secondary outcomes were modes of birth (cesarean section, episiotomy, and instrumental delivery), neonatal Appearance, Pulse, Grimace, Activity, and Respiration (APGAR) score. (APGAR) score and preterm birth (<37 weeks of gestations), and birth weight (<2500 or ⩾2500 g).

There were no changes after trial commencement in the outcome variables. These outcomes were collected by independent data collectors during labor and delivery (to record mode of birth, APGAR scores, immediate neonatal outcomes), shortly after birth (for neonatal weight and health status), and early postpartum period (to capture maternal and neonatal complications and early outcomes).

### Data collection

From October 2023 to December 2023, midwives at each participating hospital collected baseline data from study participants. The base line date were collected by research team midwives including: the socio-demographic information: mothers’ age, residence, occupation, educational status, marital status, religion, obstetric and gynecological history; parity, gravity, abortion history, contraceptive history, history of still birth age at first marriage, age at first pregnancy, whether the pregnancy was wanted and planned, and mothers’ medical history (history of diabetes mellitus, gestational hypertension, and cardiac diseases), through in-person interviews. During the study period, information on maternity unit service utilization was gathered. Data related to ANC was obtained from the maternal registry, while maternal and neonatal outcomes, including postnatal care utilization, were collected by an independent data collector who was blinded to the intervention allocation. Maternal and neonatal outcome data, including early-postnatal care utilization, were collected by an independent data collector who was blinded to intervention allocation.

These independent collectors (midwives) used standardized checklists and gathered data from medical records and maternal interviews during the early postnatal period. All details of the pregnant women were de-identified to ensure that individuals could not be identified. The data collection tools were structured, pre-tested, and initially prepared in English, then translated into the local language (Sidamaic Afaoo) by a language expert. Six midwives were recruited as data collectors, and two supervisors with master’s degrees in clinical midwifery were appointed to implement consistent monitoring across each site. The data collectors received 2 days of training to familiarize them with the tools and ensure they clearly understood the study’s objectives.

### Data analysis

The dataset was obtained from the Kobo Toolbox server and then exported to Microsoft Excel 2013. Subsequently, it was imported into STATA version 16 is a general-purpose statistical software package developed and distributed by StataCorp LLC for data cleaning, recording, and analysis. Descriptive statistics were utilized to summarize the sociodemographic and obstetric characteristics of the study participants. Pearson’s chi-square tests were used to evaluate differences in pregnancy outcomes between the intervention (MLCC) and the shared care group. Multinominal regressions were used to analyze the mode of birth categorized into three types. This method allowed us for estimation of the effects of various predictors on the likelihood of each birth category relative to a reference group, thus providing insights into factors influencing delivery outcomes. Logistic regression was used to determine the predictors for outcome variables.

To assess the effect of MLCC compared to the shared care on the outcomes, multivariable analyses using generalized linear models (GLMs) for binary outcomes with a log link were performed to estimate adjusted risk ratios (aRR) and 95% CIs. Mother’s age, parity, gravida, age at first pregnancy, age at first marriage, pregnancy planned, pregnancy wanted, residence, mother’s occupation, mother’s education, and marital status were included in GLMs for adjusting. The variables were selected based on their established influence on birth outcomes.^24-26^ These factors are known to correlate with maternal and neonatal health, ensuring a more accurate assessment of the intervention’s effects. Data analysis was carried out using STATA version 16, with *p* < 0.05 considered statistically significant.

### Ethical approval and consent to participate

Ethical approvals were obtained from the Institutional Review Board of Hawassa University College of Medicine and Health Sciences ref. no. IRB/080/14, and permission was granted by the Sidama Regional Health Bureau.

Written informed consent was secured from each study participant prior to the data collection process, and consent was obtained from the parents or legal guardians of all participants under 18 years of age. This consent was obtained after explaining the study’s objectives, the data collection methods, the potential benefits and risks of participation, and the voluntary nature of involvement in the study. This study was conducted according to the Helsinki. Declaration. This study was conducted and reported in accordance with the STROBE guidelines.^24^

## Results

### Sociodemographic characteristics

There were various significant differences between the intervention and the shared care group’s demographic characteristics (see Table 1). In the MLCC group, 24.2% of the participants were below the age of 18, while in the shared care group this percentage was 23.2%. In both groups, the majority of participants lived in urban areas, but this percentage was higher in the MLCC group. In comparing the monthly income between MLCC and shared care, the results indicate notable differences in the income distributions of the two groups. The mean monthly income was 6033.90 ETB with a standard deviation of 2530.08. In terms of the mothers’ educational status, 27.1% of those in the MLCC group and 30.9% of those in the shared care group had completed college or above.

**Table 1.** Sociodemographic characteristics of study participants in Sidama region, Ethiopia, 2024.

Variable	Category	Midwifery-led continuum of care *N* (%) *N* = 236	Shared care *p* value *N* (%) *N* = 233
Age	<18 18–25 ⩾25 Mean (SD)	57 (24.2) 95 (40.3) 84 (35.6) 26.2 (5.2)	54 (23.2), 0.038 70 (30.0) 109 (46.8) 25.4 (4.8)
Residency	Rural Urban	15 (6.4) 221 (93.6)	97 (41.6), <0.001 136 (58.4)
Mother’s educational status	No formal education Primary education Secondary education College level and above	8 (3.4) 26 (11.0) 138 (58.5) 64 (27.1)	30 (12.8), <0.001 27 (11.6) 104 (44.6) 72 (30.9)
Occupation of the mother	Student Housewife Employed Others^a^	17 (7.2) 187 (79.2) 32 (12.9) 0 (0)	33 (14.2), <0.001 77 (33.0) 116 (49.8) 7 (3.0)
Ethnicity	Sidama Amhara Other^b & c^	210 (89.0) 10 (4.2) 16 (6.7)	128 (54.9),<0.001 24 (10.3) 80 (34.8)
Religion	Protestant Ethiopian, orthodox Muslim Catholic Other^d^	200 (84.7) 21 (8.9) 15 (6.3) 0 (0) 0 (0)	124 (53.2),<0.001 66 (28.3) 19 (8.2) 22 (9.4) 2 (0.9)
Monthly income in Ethiopian Birr	1000–3000 3001–5000 ⩾5001	37 (15.7) 108 (45.8) 91 (38.6)	2 (0.9) <0.001 24 (10.3) 207 (88.8)

SD: standard deviation.

a Shared care (farmer and merchant).

b Midwifery-led continuum of care (Wolayita, Oromo, Gurage, and Hadiya).

c Shared care (Gurage, Hadiya, Kambata, and Tigre).

d Shared care (only Jesus).

*p* value = x2 SD.

### Obstetrics and gynecologic characteristics of the participant’s

The mean age of women experiencing their first pregnancy in the MLCC group was 21 ± 2.8 years, while the mean age in the shared care group was 21 ± 2.3 years. In terms of pregnancy history, 208 participants (88.1%) in the MLCC group indicated that their pregnancies were planned, which is higher than the 187 participants (80.3%) reported in the shared care group. Regarding abortion history, 34 participants (14.4%) in the MLCC group reported having experienced an abortion, which is significantly higher than the 1.7% observed in the shared care group. Most participants in both groups reported a history of contraceptive use, with 157 (66.5%) in the MLCC group and 187 (80.3%) in the shared care group (see Table 2).

**Table 2.** Obstetrics and gynecologic characteristics of study participants in Sidama region, Ethiopia, 2024.

Variable	Category	Midwifery-led continuum of care (*N* = %) *N* = 236	Shared care *p* value (*N* = %) *N* = 233
Pregnancy wanted	Yes No	208 (88.1) 28 (11.8)	187 (80.3), 0.001 46 (19.7)
Pregnancy planned	Yes No	197 (83.5) 39 (16.5)	181 (77.7), 0.037 52 (22.3)
Contraceptive use	Yes No	157 (66.5) 79 (33.5)	187 (80.3), 0.001 46 (19.7)
Abortion history	Yes No	34 (14.4) 202 (85.6)	4 (1.7), 0.001 229 (98.3)
Age at first marriage	16–18 19–21 22–24 ⩾25 Mean (± SD)	77 (32.6) 98 (41.5) 38 (16.1) 23 (9.7) 20.1 (2.7)	91 (39.1), 0.021 77 (33) 55 (23.6) 10 (4.3) 19.9 (2.5)
Gravidity	Primigravity Multigravida	97 (41.1) 139 (58.9)	114 (48.9), 0.622 119 (51.1)
Age at first pregnancy	16–18 19–21 22–24 ⩾25 Mean (± SD)	45 (19.1) 106 (44.9) 51 (21.6) 34 (14.4) 21.1 (2.8)	19 (8.2), 0.032 112 (48.1) 83 (35.6) 19 (8.2) 21.4 (2.3)

### Intervention implementation

The number of participants who had four or more ANC visits was 227 (96.2%) in the MLCC group compared with shared care group 25 (10.7%). The number of participants who had ANC visits by the same midwives was 229 (97%) in the MLCC group compared with 23 (9.9%) in shared care group. During labor, the majority of women (94.1%) in the MLCC group received intrapartum care from their assigned or backup midwives at least once, compared to only 7.3% in the shared care group. Additionally, 88.5% of women in the MLCC received postnatal care from the same midwives who provided care during pregnancy, labor, and delivery, while this percentage was significantly lower (20.2%) in the shared care group (see Table 3).

**Table 3.** Study participants intervention exposure on continuity of care in Sidama region, Ethiopia, 2024.

Variable	Category	Midwifery-led continuum of care *N* = 236 (%)	Shared care *p* value *N* = 233 (%)
Number of ANC visits	One Two Three Four or more	2 (0.8) 4 (1.7) 3 (1.3) 227 (96.2)	155 (66.5), 0.001 24 (10.3) 29 (12.4) 25 (10.7)
The same midwives at each ANC	Yes No	229 (97) 7 (3)	23 (9.9), 0.001 227 (90.1)
Frequency of ANC visits provided by the same midwives	Once Two to three times Four or more times	– 12 (5.1) 224 (94.9)	49 (21), 0.001 157 (67.3) 27 (11.6)
Had met at least one of the midwives providing care during labor at least once during their ANC visits	Yes No	222 (94.1) 14 (5.9)	17 (7.3), 0.001 216 (92.7)
Had met the midwife who provided their intrapartum care during their early postnatal period	Yes No	209 (88.5) 27 (11.5)	47 (20.2), 0.001 186 (79.8)
Number of PNC visit	Once Two to three times Four or more	176 (74.6) 60 (25.4)	222 (95.3), 0.001 9 (3.9) 2 (0.9)

ANC: antenatal care; PNC: postnatal care.

### Obstetric complications

**Figure 2.**
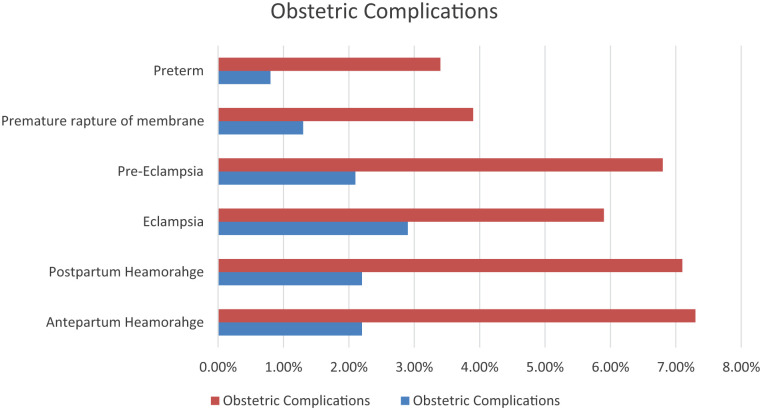
Obstetric complications by study participants, from October 2023 to June 2024, Sidama regional sate, Ethiopia.

Antepartum hemorrhage occurred in 2.2% of the MLCC group, compared to 7.3% in the shared care group (*p* = 0.003). Eclampsia occurred in 2.1% of women in the MLCC group, which is significantly lower compared to the shared care group where it was reported in 9% of women (*p* = 0.006). There were three perinatal deaths (two still birth, and one early neonatal death), and all occurred among women in the shared care group (see Figure 2).

### Maternal and newborn outcomes

In the MLCC group, women experienced spontaneous vaginal births, compared to in the shared care group, with a significant adjusted risk ratio (aRR) of 1.21 (95% CI 1.14–1.67). Additionally, compared to the shared care group, the study found that women in the MLCC group had significantly lower rates of cesarean sections, vacuum-assisted births, and episiotomies by 67.2%, 61.5%, and 41.8%, respectively, compared to women in the shared care group. Women who received MLCC were further significantly less likely to have preterm newborns compared to those in the shared care group, with an aRR of 0.16 (95% CI 0.11–0.57), with the attribute risk percentage of 75.8% (95% CI 11.2–93.4), indicate 75.1% of the difference having preterm can be attributed to the exposure to the MLCC model.

Additionally, women in the MLCC group were more likely to give birth to newborns weighing 2500 g or more, with an adjusted relative risk (aRR) of 0.46 (95% CI 0.33–0.79) with the attribute risk percentage of 66.1% (95% CI: 36.2–82), indicate 66.1% of the difference in the likelihood of having newborns with birth weight ⩾2500 g can be attributed to the exposure to the MLCC model.

The incidence of low birth weight in the MLCC group was three cases, corresponding to a mean incidence of 1.27%. In contrast, the shared care group recorded 12 cases, with a mean incidence of 5.15%. The mean difference in the incidence of low birth weight between MLCC and shared care is 3.8. This indicates that the incident of LBW is lower in MLCC compared to the shred care. Additionally, the predictive model estimated indicated that only 1.27% of newborns in MLCC would have a birth weight <2500 g. This suggests a low risk of low birth weight in this group, indicating positive birth weight outcomes under MLCC.

The study estimated that the proportion of preterm in newborns could be significantly lower by 84% among women allocated to the MLCC group compared to those in the shared care group (see Table 4).

**Table 4.** The effect of midwifery-led continuum of care model on maternal and newborn outcomes in Sidama region, Ethiopia, 2024.

Variables	Categories	Midwifery-led continuum care *N* = 236	Shared care *N* = 233	Unadjusted risk ratio^a^ (95% CI)	Adjusted risk ratio^b^ aRR (95% CI)	Attributable-risk percentage (95% CI)
SVD	Yes No	209 (88.5%) 27 (11.5%)	146 (62.7%) 87 (37.3%)	1.09 (1.03–1.10)	1.14 (1.07–1.34)	59.7 (43.3–71.4)
Birth weight	⩾2500 g <2500 g	228 (96.6%) 8 (3.4%)	197 (84.5%) 36 (15.5%)	1.01 (1.03–1.14)	0.46 (0.33–0.79)	66.1 (36.2–82)
Vacuum	Yes No	5 (2.1%) 231 (97.8%)	20 (8.6%) 213 (91.4%)	0.391 (0.18–0.51)	0.385 (0.21–0.69)	61.5 (15.4–82.5)
C/S	Yes No	2 (0.9%) 231 (86%)	33 (14%) 203 (99.1%)	0.28 (0.15–0.73)	0.32 (0.19–0.57)	89.2 (58.6–97.2)
Episiotomy	Yes No	15 (6.3%) 221 (93.6)	34 (14.6%) 199 (85.4%)	0.44 (0.17–0.93)	0.59 (0.27–0.79)	41.8 (10.4–62.2)
PROM	Yes No	8 (3.4) 228 (96.6%)	20 (8.6%) 213 (91.4%)	0.39 (0.17–0.87)	0.36 (0.14–0.93)	57.0 (20.2–76.8)
Preterm	Yes No	2 (0.8%) 234 (99.1)	14 (6%) 219 (93.9)	0.14 (0.03–0.61)	0.16 (0.11–0.57)	75.8 (11.2–93.4)
APGAR at fifth	Yes No	1 (0.4%) 235 (99.6%)	4 (1.7%) 229 (98.2%)	0.31 (0.26–0.53)	0.37 (0.20–0.47)	28.9 (14.2–40.9)

APGAR: appearance–pulse–grimace–activity–respiration; CI: confidence interval; C/S: cesarean section; PROM: premature rapture of membrane; SVD: spontaneous vaginal delivery.

a Univariate analyses using Pearson’s chi-squared tests.

b Multivariable analyses using generalized linear models for binary outcomes with the log link.

## Discussion

The study examined the effects of the MLCC model compared to shared care group, on maternal and neonatal health outcomes in the Sidama region in Ethiopia. The findings from this study revealed that the MLCC model had many benefits compared to shared care group, showing significantly higher rates of spontaneous vaginal birth, lower rates of cesarean sections, lower rates of episiotomy and instrumental vaginal births, less premature rupture of the membrane, and less occurrence of preterm births and low-birth weight.

Being assigned to MLCC was associated with an increased likelihood of spontaneous vaginal deliveries, while decreasing the rate of emergency cesarean sections and vacuum-assisted birth. Studies conducted in high-income countries likewise indicated that women who underwent MLCC were more prone to experience spontaneous labor,^9–11,16,27–30^ and were less likely to have an elective cesarean section.^10,11^ Previous systematic reviews conducted in low-income countries also support the current findings.^9,17,30^ This study further revealed that women assigned to the MLCC model experienced a lower episiotomy rate compared to those in shared care group women. Similar findings were reported in some other studies as well.^10,11,15,28,30^ A possible explanation of the positive findings could be that MLCC emphasizes the importance of women’s well-being and the promotion of natural childbirth by optimizing their physiological ability to give birth with minimal or no interventions.^10,31^ Furthermore, woman’s confidence and comfort during labor, supported by a familiar midwife, may reduce the likelihood of medical interventions, such as emergency cesarean sections.^10,28,32^ Episiotomies specifically are a contentious issue in many developing countries, where they are often conducted routinely, even in situations where they may not be medically necessary, and the women are not being asked for consent prior to the procedure.^33,34^ Episiotomies should be carried out by midwives and only with the patient’s consent.^35,36^

When women receive care through the MLCC approach, the midwives develop a stronger rapport with their patients. This increased familiarity enables more in-depth discussions about the delivery plan and potential interventions. As a result, a woman’s unique needs and preferences are given heightened consideration and attention,^10^ including discussing her thoughts on options for assisted birth, such as episiotomies.

However, in contrast to current and previous findings, studies from Iran, Ireland, and Singapore found no statistically significant difference in mode of birth between the MLCC and the shared care group.^29,37–39^ A possible reason could be discrepancies between the health care systems, including substantial differences in financial resources, access to advanced technologies, development of health care infrastructure, availability of well-trained professionals, and investment in social services that address social determinants of health.^29,37,39^

In the current study, the rate of preterm birth was significantly lower in MLCC as compared to the women who received shared care. This finding was in line with previous studies,^17^ including studies from Australia and Palestine.^21,28,38,40,41^ This study further revealed that neonates born to women assigned to the MLCC group had a significantly lower rate of low 5-min APGAR scores (below 7) compared to neonates of the shared care group. This finding aligns with the results of a Cochrane review.^10,15^ However, the findings contradict a study conducted in the Ireland that no statistically significant difference in 5-min APGAR scores between the MLCC and shared care group.^39^ One possible explanation could be the disparities in health care systems, as the Irish healthcare system features advanced medical care, highly skilled health professionals, comprehensive prenatal and postnatal services, and substantial investment in health infrastructure, in contrast to the Ethiopian health care system.^39^ Furthermore, this study revealed that women assigned to the MLCC had neonates with better birth weight (>2500 g) compared to neonate in the shared care group. These findings were in line with a study conducted in Canada.^41^ The current study further found that women receiving MLCC more often had four or more ANC visits compared to those in shared care group.^15^ A possible explanation could be that in MLCC relationships based on trust, confidence, and advocacy are established, resulting in women experiencing greater safety, reduced stress, and increased feelings of security and respect. This might also encourage them to seek and participate in ANC, providing more chances for early prevention and diagnosis of complications,^42^ hence preventing preterm birth. Additionally, women in the MLCC model maintained consistent engagement with their healthcare providers throughout their pregnancies. This continuity not only increases the likelihood of attending the recommended number of ANC visits but also contributed to a more positive birth experience, resulting in higher rates of spontaneous deliveries.

This study implies that the implementation of MLCC has a positive effect on maternal and neonatal health outcomes, highlighting the crucial role that midwives play in improving maternal healthcare. Facilitating midwives’ practice of MLCC can greatly enhance the quality of maternal health services in Ethiopia. By tackling the challenges that midwives encounter and establishing a supportive framework, the care they provide can be improved, resulting in better health outcomes for both mothers and newborns.

However, the absence of a supportive system for midwives to practice MLCC in Ethiopia hampers the quality of care they are able to deliver.^15^ In Ethiopia, maternity care has traditionally been fragmented, involving multiple health care providers including hospital-based midwives, nurses, general practitioners, and obstetricians. This division of responsibilities can lead to inconsistencies in care, as communication between different providers may be inadequate, resulting in gaps in service delivery and continuity of care for mothers and newborns.^43^

### Strengths of the study

This study is the first to provide evidence on maternal and neonatal outcomes for women who received continuous care from the same midwife throughout their pregnancy, utilizing a MLCC model tailored for the Sidama region in Ethiopia. Additionally, the study experienced minimal loss to follow-up (*n* = 9), and the combination of medical record reviews and interviews with mothers enhances the reliability of the assessment of intervention outcomes. Furthermore, this study used clear inclusion and exclusion criteria to recruit participants and used a prospective approach. The findings could inform policy discussions about this alternative approach in maternal and newborn health. Additionally, the clear eligibility criteria help create a more homogenous study population, reducing confounding variables and making it easier to attribute outcomes to MLCC.

### Limitations of the study

The study’s present evidence of the model is limited to low-risk women only, as high-risk women were excluded from the study. Also, although 469 women were included in the study, this sample is too small to identify differences in rare outcomes between the intervention and control groups. The collection of sociodemographic, obstetric, and gynecological history depended on self-reported measures, which may introduce biases such as social desirability and recall bias. Additionally, since midwives were recruited based on their willingness to participate, this can introduce biases that impact the validity of the study’s findings, making it difficult to reach accurate conclusions regarding the effectiveness of the MLCC model.

Furthermore, the differences in characteristics between the intervention and control groups in MLCC can introduce biases that affect the reliability and generalizability of the findings. A key limitation of this study is the absence of randomization. This makes it vulnerable to selection bias, where personal preferences in choosing care models can significantly affect outcomes. The lack of randomization also complicates the control of confounding variables, such as socioeconomic status and preexisting health issues, thereby threatening the accurate assessment of the intervention’s impact. However, we have adjusted the multivariate models in the statistical analyses for potential confounding variables. Nonetheless, potential biases include systematic differences between care groups, and performance bias resulting from variations in care delivery, such as midwives offering more personalized care.

Another limitation stems from using a lottery method to select the first participant in each hospital. This approach risks selection bias if the list of eligible participants is incomplete, potentially excluding a specific subgroup. Additionally, the lack of blinding during recruitment, where midwives explained the study aims, could have led to conscious or unconscious selection bias. We acknowledge that an error occurred during the sample size calculation. The discrepancy between the intended and actual sample size resulted in a sample that was 11 participants larger than originally.

## Conclusion

The findings from this study suggest that implementing a MLCC model associated with improved maternal and neonatal health outcomes in the low-resource Sidama region of Ethiopia. Women who received MLCC appeared to have higher rates of spontaneous vaginal birth, and lower rates of cesarean section, episiotomy, instrumental vaginal birth, premature rupture of membranes, preterm birth, and low birth weight compared to those receiving usual care in the shared care group. Further research may help to clarify the practical application of such care models, particularly for women facing social risk factors or high-risk pregnancies in low-resource settings, and explore the potential value of integrating new technologies.
